# Malignant Transformation of a Borderline Ovarian Tumor With Pulmonary and Pleural Metastases After Years of Latency: A Case Report and Literature Review

**DOI:** 10.3389/fmed.2020.571348

**Published:** 2020-09-30

**Authors:** Jiang-wei Ma, Yuan Miao, Chao-nan Liang, Ning Wang, Bin Jiang, Qiu-yue Wang, Jian Kang, Gang Hou, Yan Yin

**Affiliations:** ^1^Department of Pulmonary and Critical Care Medicine, First Hospital of China Medical University, Shenyang, China; ^2^Department of Pathology, The First Hospital and College of Basic Medical Sciences, China Medical University, Shenyang, China; ^3^Gynecology Department, The Second Affiliated Hospital, Dalian Medical University, Dalian, China; ^4^Department of Ultrasound, First Hospital of China Medical University, Shenyang, China

**Keywords:** metastasis, pulmonary cystic nodules, transbronchial lung biopsy, pleural biopsy, borderline ovarian tumor

## Abstract

Borderline ovarian tumor (BOT) refers to a distinct tumor of the ovary of epithelial origin and typically has a favorable prognosis. However, these tumors are not exempt from risks of recurrence and malignant transformation, which can arise from the remaining ovarian tissue, peritoneal implants, or distant localization. Here, we report a case of a mucinous BOT with multiple pulmonary cystic nodules without evidence of pulmonary metastasis even after two fine needle biopsies. Staging surgery was performed, and no evidence of peritoneal implants or invasion to adjacent organs found. At the end of the 7-year monitored follow-up after surgery, the pulmonary lesions were found to be increased in size. The transbronchial lung biopsy and pleural biopsy confirmed transformation into malignant mucinous adenocarcinoma with pleural metastasis. In the current case, we observed potential pulmonary metastasis of the BOT with malignant transformation and a latency as long as 7 years, which reminds us that multiple pulmonary cystic changes in patients with BOTs should be screened carefully to evaluate the pulmonary involvement of BOTs and potentially false-negative results after fine needle biopsy. Thus, a thorough check-up for complete staging of the disease and a close long-term follow-up to monitor potential recurrence and malignant transformation are advised.

## Introduction

Borderline ovarian tumor (BOT) refers to a group of ovarian epithelial tumors officially defined by the International Federation of Gynecology and Obstetrics (FIGO) in 1961 and by the World Health Organization (WHO) in 1973. Histopathologically, the behavior of this group of neoplasms is intermediate between the behavior of benign cystadenomas and invasive carcinomas; however, the morphological changes are diverse, and the behavior is therefore heterogenous. The serous and mucinous subtypes are the most common forms (1). Although BOTs have a favorable prognosis, an advanced FIGO stage, recurrence, and malignant transformation are critical factors affecting the prognosis. Here, we report a case of a mucinous BOT with multiple pulmonary cystic nodules without obvious evidence of pulmonary metastasis even after two fine needle biopsies. During the careful 7-year follow-up after cytoreductive surgery, the pulmonary lesions were proven to be pulmonary metastases that had transformed into malignant mucinous adenocarcinoma and pleural metastasis.

## Case Presentation

A 53-year-old female was referred to our department with complaints of a slight dry cough and enlarged pulmonary nodules [computed tomography (CT) value: 15 Hounsfield units (HU)] with slight peripheral enhancement, which were under close monitoring after cytoreductive surgery for a BOT.

**Figure 1:**
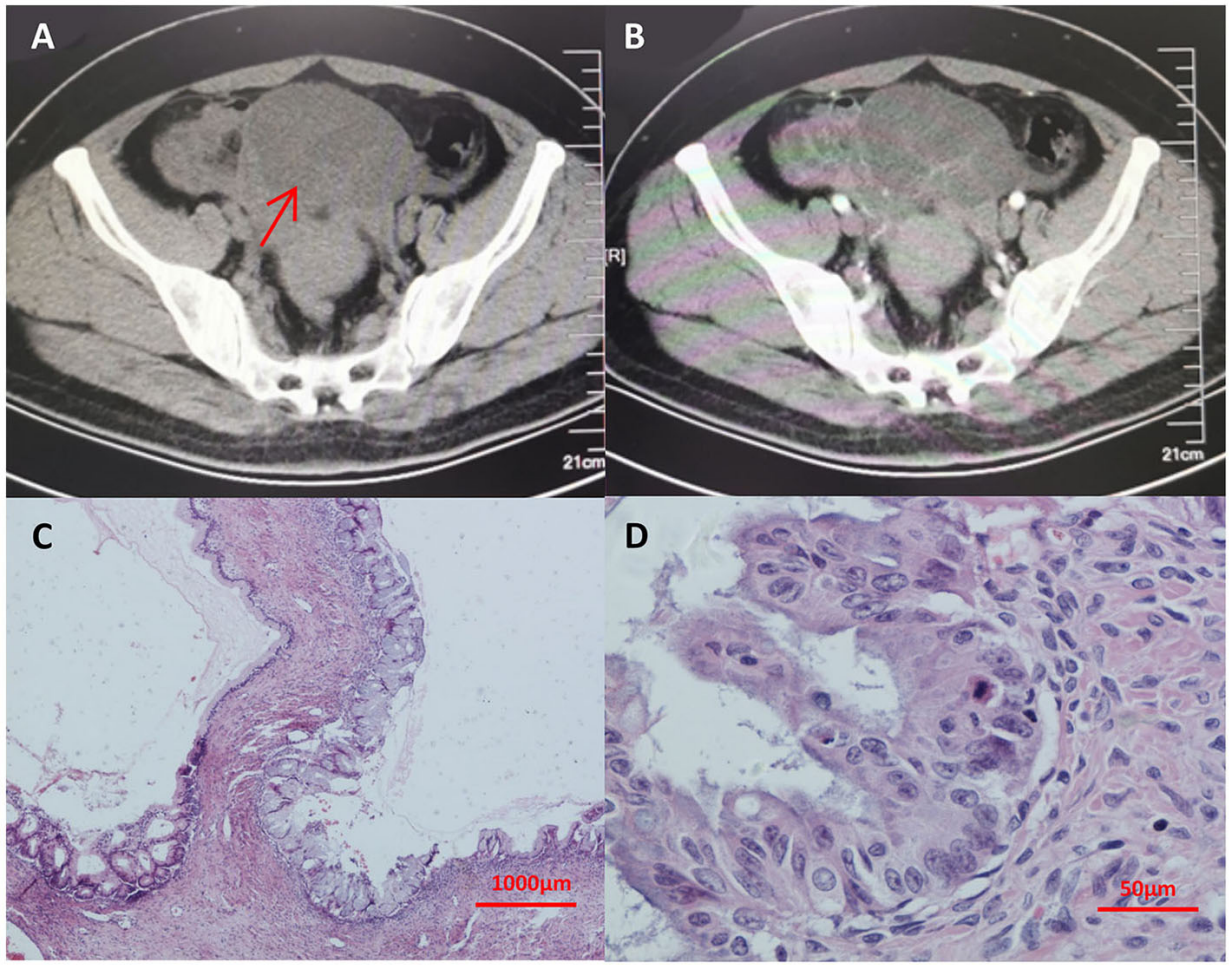
**(A,B)** Abdominal enhanced CT showing a giant pelvic-abdominal cyst with an uneven density (7 HU) with peripheral and internal septal enhancement. The giant cyst extended from the pelvic cavity to the level of right renal pelvis, measuring 20 cm × 40 cm, with ascites. **(C,D)** The final pathology from hematoxylin-eosin staining after laparotomy confirmed the diagnosis of ovarian mucinous borderline cystadenoma with no involvement of the left ovary, omentum, and bilateral adjacent lymph nodes. In **(A)**, arrow indicates the giant pelvic-abdominal cyst with an uneven density (7 HU) with peripheral and internal septal enhancement. CT, computed tomography.

**Figure 2:**
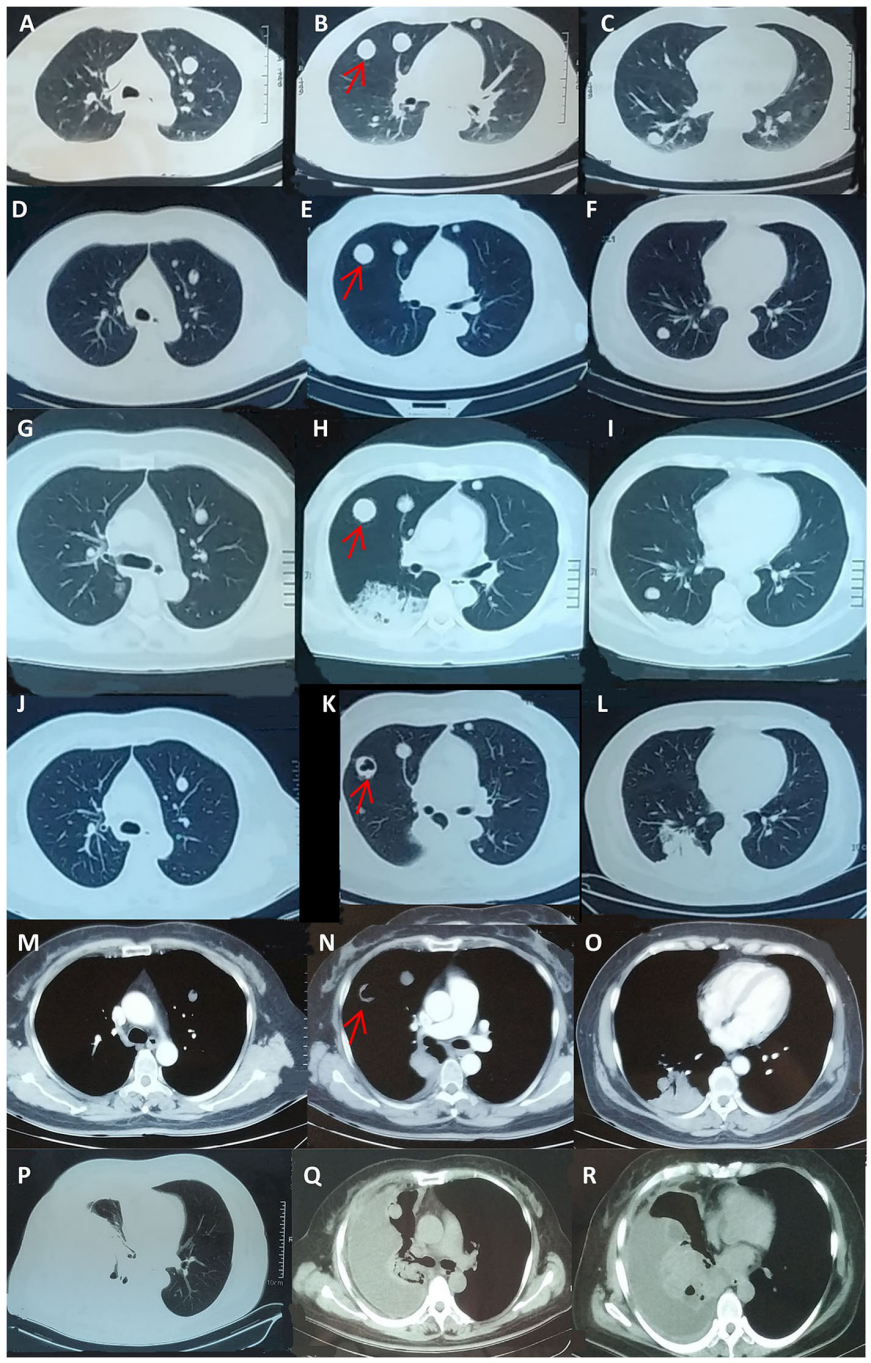
Follow-up chest CT scans during seven-year monitoring of the pulmonary nodules. **(A–C)** Detection of the bilateral pulmonary nodules before laparotomy. **(D–F)** The chest CT scan at the sixth year after laparotomy; the patient was asymptomatic. **(G–O)** At the seventh year of the follow-up, the patient was referred to the respiratory department and presented with cough and clear sputum without hemoptysis or any other symptoms. Chest CT showed a new irregular nodule at the dorsal segment of the right inferior lobe, and a cavity was observed in the largest original pulmonary nodule. **(P–R)** After four cycles of chemotherapy, the pulmonary solid metastasis progressed with the development of pleural effusion and pleura thickening, which was later proven to be metastatic adenocarcinoma by pleural biopsy. The patient passed away 4 months later. The arrows indicate one pulmonary nodule with progress during the follow up of 7 years respectively. CT, computed tomography.

Seven years prior, she had suffered from progressive abdominal distention. Abdominal contrast-enhanced CT (Figures 1A,B) and a thorough check-up revealed a giant pelvic-abdominal cyst with an uneven density (7 HU) with peripheral and internal septal enhancement. The giant cyst extended from the pelvic cavity to the level of right renal pelvis, measuring 20 × 40 cm, with ascites. Meanwhile, chest CT showed multiple bilateral cystic nodules without obvious enhancement (Figures 2A–C). The patient's serum carbohydrate antigen 125 (CA-125) and CA-19-9 levels were increased to 69.15 U/ml (0–35 U/ml) and 369.4 U/ml (0–37 U/ml), respectively. Other serum tumor markers [carcinoembryonic antigen (CEA), alpha-fetoprotein (AFP), neuron-specific enolase (NSE), and cytokeratin 19 fragments] were within normal ranges. For final diagnosis of the mass in the abdomen, exploratory laparotomy was planned. Before laparotomy, to determine the nature of the nodules in the lungs and the detailed staging of the mass, the patient underwent percutaneous biopsy, and the histopathological results indicated only fibrosis and degeneration of the lung tissue but no evidence of metastasis from the ovary. Repeated percutaneous biopsy for another nodule was performed to avoid a missed diagnosis of metastasis, and the histopathology confirmed the previous results showing clear cystic fluid but not metastasis. Subsequent laparotomy revealed a cystic mass with a smooth surface and an intact capsule on the right ovary and ascites. Intraoperative frozen section revealed an ovarian mucinous borderline cystadenoma (MBOT). Exfoliative cytological examination revealed no evidence of implantation in the ascites. Thus, staging surgery (bilateral salpingo-oophorectomy (BSO), hysterectomy, appendectomy with omentectomy, and the adjacent lymphadenectomy including para-aortic lymph nodes, and pelvic lymph nodes) was performed. The final pathology confirmed the diagnosis of ovarian mucinous borderline cystadenoma, intestinal type, with the intestinal epithelium rich in goblet cells and the stratification of cells showing light to moderate atypia in a papillary structure (Figures 1C,D) with no involvement of the left ovary, omentum, and bilateral adjacent lymph nodes. Thus, the FIGO stage at diagnosis was incomplete stage due to the obscure diagnosis of the lung nodules. After resection of the tumor, the serum CA-125 and CA-19-9 levels of the patient declined to normal values of 27.5 and 18.09 U/ml, respectively, which is consistent with the reported correlation between preoperative CA-125 levels and increasing FIGO stages of BOTs. Then, the patient was followed closely for the pulmonary nodules on CT scans (Figures 2D–F) and for possible asymptomatic recurrence of the BOT and malignant transformation arising from the BOT.

During the following 7 years, CA-125 was within the normal range after the risk-reducing cytoreductive surgery, and the size and morphology of the pulmonary nodules were stable.

**Figure 3:**
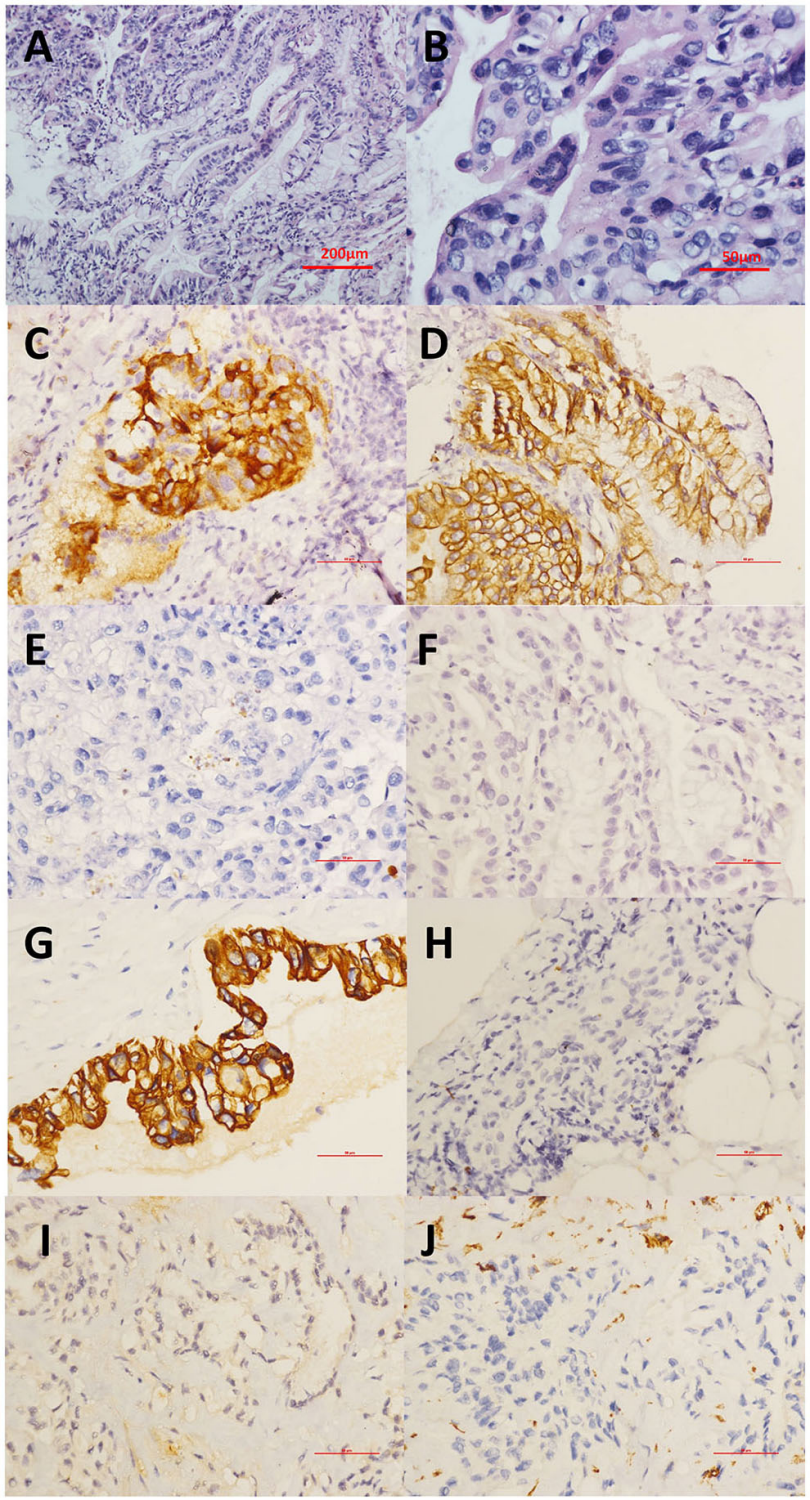
**(A,B)** Transbronchial lung biopsy (TBLB) targeting the new consolidation was performed. The histopathology of the HE staining result indicated papillary mucinous adenocarcinoma. **(A)** This tumor demonstrates architectural complexity with characteristics of mucinous differentiation. **(B)** High-power image highlighting prominent epithelial stratification and cytologic atypia. **(C–F)** Immunostaining suggesting papillary mucinous adenocarcinoma as the malignant transformation arising from the BOT with **(C)** CA-125(focal+), **(D)** CK7(+), **(E)** TTF-1(–), and **(F)** CDX2(–). **(G–J)** Immunohistochemical staining of the pleural biopsy showed **(G)** CK7(+), **(H)** TTF1(-), **(I)** CR (–), and **(J)** VIM (–). CA-125, Carbohydrate antigen 125; CDX2, Caudal type homeobox transcription factor 2; CK7, Cytokeratin 7; CR, Complement receptor; HE, hematoxylin-eosin; TTF-1, Thyroid transcription factor-1; VIM, Vimentin.

For this admission in our department at the end of the seventh year during the monitoring of the disease, the patient presented with cough and clear sputum without hemoptysis or any other symptoms. Chest CT showed a new irregular nodule at the dorsal segment of the right inferior lobe, and a cavity was observed in the largest original pulmonary nodule (Figures 2G–O). The patient's serum CA-125 and CEA levels were elevated to 68.9 and 66.50 U/mL (normal range 0 ~6 U/ml), respectively. Abdominal CT scan revealed no obvious abnormality or ascites. Thus, transbronchial lung biopsy (TBLB) targeting the new consolidation was performed. The histopathology result indicated papillary mucinous adenocarcinoma, while immunostaining suggested malignant transformation arising from the BOT [Cytokeratin(CK)(+), P63(–), CK5/6(–), Napsin-A(–), Transcription Factor-1(TTF-1)(–), Cluster of Differentiation(CD)56(–), Ki-67(15%), CA-125(focal+), Estrogen receptor (ER)(–), paired box gene 8(PAX8)(–), CK7(+), CK20(–), Caudal type homeobox transcription Factor 2(CDX2)(–), and P16(–)] (Figures 3A–F). Breast cancer susceptibility gene (BRCA) mutation was not observed in the tissue of the lung biopsy. After four cycles of chemotherapy [AUC (area under the curve): 5 mg/ml/min of carboplatin and 65 mg/m^2^ of docetaxel per cycle] with 3-week intervals were completed, the pulmonary solid metastasis progressed with the development of pleural effusion and pleura thickening (Figures 2P–R). Ultrasonic-guided transcutaneous pleural biopsy revealed metastatic adenocarcinoma (Figures 3G–J). The patient passed away 4 months later.

## Discussion

Here, we report a case of malignant transformation 7 years after the resection of a BOT and normalized serum CA-125 after the surgery, which presented as invasive pulmonary and pleural malignant transformation of the BOT into papillary mucinous adenocarcinoma. To our knowledge, this is the first case report of malignant transformation arising from a stable pulmonary BOT, which was missed by the initial fine needle biopsies, with no progress of the primary residual lesion.

BOTs account for approximately 10–20% of all ovarian neoplasms (2), with an incidence of 1.8–4.8/100,000 females per year (3). As the name of BOTs indicates, their behaviors are not always entirely benign. Although BOTs typically have a favorable prognosis, with excellent 5-, 10-, 15-, and 20-year survival rates as high as 97, 95, 92, and 89%, respectively (4), they are still challenging for clinicians due to several complexities: most tumors present at an early stage, but their diagnosis is often determined only after surgery; thus, the heterogeneity of surgical management is complicated. BOTs present a diverse morphology, resulting in heterogenous behaviors that are complicated for clinicians to predict. Despite the good prognosis, these tumors are not exempt from the risks of recurrence and malignant transformation (5).

The pathological diagnosis of BOTs is complicated. A significant proportion of cases diagnosed as BOTs on frozen section will be classified as malignant on final pathology, especially when lymphadenectomy is not undertaken or when fertility-sparing surgery is performed. In addition, interobserver variations in the histopathological reporting of BOTs emphasize the significance of central pathological review by an expert gynecological pathologist. In some cases, ovarian malignancies were initially diagnosed as BOTs based on equivocal original pathology, but follow-up examinations revealed no BOT-precursor malignancies such as serous primary peritoneal carcinoma and Sertoli-Leydig tumor (5). Thus, complete staging, pathological review, and BOT-related evidence are significant for the diagnosis of malignant transformation of BOTs. In our case, the final pathology of both the lung biopsy and the pleural biopsy confirmed the pulmonary and pleural malignant transformation by immunostaining demonstrating BOT-induced markers. Initially, however, the pulmonary fluid-containing cystic nodules were misdiagnosed twice by cytological examination via fine needle biopsy. For complete staging of BOTs with pulmonary cystic nodules, further examinations, for example, video-assisted thoracic surgery (VATS), might be considered for absolute confirmation of the pathological diagnosis of the pulmonary nodules. For our case, if VATS was performed, the staging might change to stage IV and the further treatment such as chemotherapy might be initiated. However, such examinations might increase the potential risk for the non-high-risk population. Besides, biological behavior and different malignancy of BOTs differs, thus the pulmonary nodules in our case could maintain stable for 7 years without obvious changes.

Recurrence rates vary in different studies (1). A systematic review (6) showed that 37% of recurrences are diagnosed during the first 2 years, 31% in years 2–5, and 32% more than 5 years after diagnosis, including 10% after more than 10 years. In different retrospective cohort studies, recurrent or persistent BOTs and malignant transformation following initial treatment were noted in 4 ~20% of patients, with a median progression-free survival (PFS) time of 14 months (1 ~36 months) (5, 7). Thus, close long-term follow-ups are essential for patients. BOT has been described as a precursor of ovarian carcinoma. Relapse can be diagnosed in remaining ovarian tissue, peritoneal implants or distant metastases. The overall rate of malignant relapse was 2.3 ~3% in a case series (5, 8). The risks of recurrence and malignant transformation are highly variable between studies corresponding to the diverse biological behaviors, with reported rates ranging from 0 ~58% and a mean rate of 3% (5). BOT recurrence has been found to occur as late as 23 ~25 years after initial diagnosis, but due to the high heterogeneity and relatively low occurrence of malignant transformation, no median time to malignant transformation has been reported. A unique feature of our case is that despite the initial pulmonary involvement of the BOT, the time to malignant transformation was 7 years. However, not all cases diagnosed as invasive ovarian malignancy after initial treatment for a BOT can be easily classified as recurrent disease or malignant transformation arising from the BOT. An advanced FIGO stage has been found to be the most prominent prognostic factor, with elevated preoperative serum CA-125 and the presence of micropapillary features as risk factors for an advanced stage at presentation (5, 7). In our case, the patient's advanced age and elevated preoperative CA-125 level and pulmonary involvement of the BOT predicated a high likelihood of malignant transformation. To monitor malignant transformation or malignancy recurrence, transvaginal ultrasound or serum CA-125 measurement should be carried out during the follow-up. In our case, although no residual recurrence was noted in the abdomen, progression of the pulmonary lesions and CA-125 elevation were observed. Thus, evaluation of the nodule size, which might be related to BOTs according to the RECIST (Response Evaluation Criteria in Solid Tumors) guideline (9), may be helpful to monitor malignant transformation or malignancy recurrence. In one retrospective review, age ≥40 years, a late stage at diagnosis and incomplete surgery were significantly associated with invasive recurrence, while complete tumor excision and a prolonged follow-up were advised for cases in which malignant transformation may occur (1).

## Conclusion

Pulmonary cystic changes in patients with BOTs should be screened to evaluate the pulmonary involvement of the BOTs. A thorough check-up for complete staging of the disease and a close long-term follow-up to monitor potential recurrence and malignant transformation are advised.

## Data Availability Statement

All datasets generated for this study are included in the article/supplementary material.

## Ethics Statement

Ethical review and approval was not required for the study on human participants in accordance with the local legislation and institutional requirements. The patients/participants provided their written informed consent to participate in this study. Written informed consent was obtained from the individual(s) for the publication of any potentially identifiable images or data included in this article.

## Author Contributions

GH and YY made substantial contributions to the conception and design of the work. YM, C-nL, and Q-yW helped to collect the data of the case. GH, YY, and J-wM write the manuscript. J-wM, GH, and YY carried out interpretation of data for the work. NW performed the laparotomy. GH, J-wM, BJ, and JK performed the transbronchial lung biopsy and pleural biopsy. All authors contributed toward acquisition of data for the work. All authors revised the paper critically for important intellectual content. All authors carried out final approval of the version to be published. All authors agree to be accountable for all aspects of the work in ensuring that questions related to the accuracy or integrity of any part of the work are appropriately investigated and resolved.

## Conflict of Interest

The authors declare that the research was conducted in the absence of any commercial or financial relationships that could be construed as a potential conflict of interest.

## Abbreviations

AFP: alpha-fetoprotein
AUC: area under the curve
BOT: Borderline ovarian tumor
BRCA: Breast cancer susceptibility gene
BSO: bilateral salpingo-oophorectomy
CA-125: carbohydrate antigen 125
CD: Cluster of Differentiation
CDX2: Caudal type homeobox transcription Factor 2
CEA: carcinoembryonic antigen
CK: Cytokeratin
CT: computed tomography
ER: Estrogen receptor
FIGO: International Federation of Gynecology and Obstetrics
HU: Hounsfield units
MBOT: ovarian mucinous borderline cystadenoma
NSE: neuron-specific enolase
PAX8: paired box gene 8
PFS: progression-free survival
RECIST: Response Evaluation Criteria in Solid Tumors
TBLB: transbronchial lung biopsy
TTF-1: Transcription Factor-1
VATS: Video-assisted thoracic surgery
WHO: World Health Organization.
